# Targeting cholesterol-rich microdomains to circumvent tamoxifen-resistant breast cancer

**DOI:** 10.1186/bcr3063

**Published:** 2011-11-24

**Authors:** Richa Tiwary, Weiping Yu, Linda A deGraffenried, Bob G Sanders, Kimberly Kline

**Affiliations:** 1 School of Biological Sciences/A5000, University of Texas at Austin, 1 University Station, Austin, TX 78712, USA; 2 Department of Nutritional Sciences/A2703, University of Texas at Austin, 1 University Station, Austin, TX 78712, USA

## Abstract

**Introduction:**

Adjuvant treatment with tamoxifen substantially improves survival of women with estrogen-receptor positive (ER+) tumors. Tamoxifen resistance (TAMR) limits clinical benefit. RRR-α-tocopherol ether-linked acetic acid analogue (α-TEA) is a small bioactive lipid with potent anticancer activity. We evaluated the ability of α-TEA in the presence of tamoxifen to circumvent TAMR in human breast cancer cell lines.

**Methods:**

Two genotypically matched sets of TAM-sensitive (TAMS) and TAM-resistant (TAMR) human breast cancer cell lines were assessed for signal-transduction events with Western blotting, apoptosis induction with Annexin V-FITC/PI assays, and characterization of cholesterol-rich microdomains with fluorescence staining. Critical involvement of selected mediators was determined by using RNA interference and chemical inhibitors.

**Results:**

Growth-factor receptors (total and phosphorylated forms of HER-1 and HER-2), their downstream prosurvival mediators pAkt, pmTOR, and pERK1/2, phosphorylated form of estrogen receptor-α (pER-α at Ser-167 and Ser-118, and cholesterol-rich lipid microdomains were highly amplified in TAMR cell lines and enhanced by treatment with TAM. α-TEA disrupted cholesterol-rich microdomains, acted cooperatively with TAM to reduce prosurvival mediators, and induced DR5-mediated mitochondria-dependent apoptosis via an endoplasmic reticulum stress-triggered pro-death pJNK/CHOP/DR5 amplification loop. Furthermore, methyl-β-cyclodextrin (MβCD), a chemical disruptor of cholesterol rich microdomains, acted cooperatively with TAM to reduce prosurvival mediators and to induce apoptosis.

**Conclusions:**

Data for the first time document that targeting cholesterol-rich lipid microdomains is a potential strategy to circumvent TAMR, and the combination of α-TEA + TAM can circumvent TAMR by suppression of prosurvival signaling via disruption of cholesterol-rich lipid microdomains and activation of apoptotic pathways via induction of endoplasmic reticulum stress.

## Introduction

Of the estimated 207,090 new cases of breast cancer diagnosed among women in the United States in 2010, approximately 70% were ER+ [1]. Unfortunately, 40% to 50% of ER+ breast cancer patients either will not respond to endocrine therapy (that is, exhibit *de novo *resistance) or will have cancer recurrence because of acquired endocrine therapy resistance [2]. Clearly, more basic science information and different treatment regimens are needed to circumvent endocrine therapy resistance.

TAM is a selective estrogen-receptor modulator with estrogenic actions in endometrial tissue, adipose tissue, and bone, and anti-estrogenic actions in breast tissue [3]. TAM, which binds to ER-α and antagonizes ER-α actions in breast tissue, has been the mainstay of endocrine therapy in both early and advanced ER+ breast cancer patients for almost three decades. However, TAM resistance remains the major barrier for its successful application in the clinic. *De novo *and acquired resistance may occur through altered cell-signaling mediators, leading to estrogen-independent activation of ER-mediated gene expression and hormone independence [4]. Of the many events producing TAMR, aberrant overexpression of prosurvival signaling is implicated as an important contributor to both acquired and *de novo *TAMR [5,6]. TAMR cells have been shown to overexpress receptor tyrosine kinases (RTKs), such as HER-1 and HER-2, and to crosstalk with membrane-associated ER (mER), leading to nuclear estrogen-receptor (nER) dependent and independent cell proliferation in which TAM acts as an agonist [6-8].

Cholesterol-enriched lipid-raft microdomains are characterized as lateral assemblies of glycosphingolipids and cholesterol that form liquid-ordered membrane phases with detergent-resistant structures. Cholesterol-enriched domains are highly expressed in tumor cells [9,10] and provide the necessary platforms for growth factors, RTKs, and their downstream mediators, such as Akt and ERK (RTKs/Akt and RTKs/ERK complexes), to interact and crosstalk, leading to cell proliferation and survival [10,11]. Therefore, cholesterol-enriched lipid-raft domains are described as "survival pools" for promoting prosurvival and pro-proliferation pathways, both of which are targets for cancer prevention and therapy.

α-TEA, a unique small pleiotropic-acting lipid, has been shown to possess anticancer properties that are selective for cancer cells and not normal cells and that are nontoxic both *in vitro *and *in vivo *[12-24]. Mechanistic studies show that α-TEA has two major effects that are necessary and sufficient for inducing apoptosis of cancer cells: (a) activation of proapoptotic pathways including Fas receptor (FasR)/Fas ligand (Fas L), endoplasmic reticulum stress-mediated JNK/CHOP/DR5 and p73/Noxa, leading to caspase-8 and mitochondria-dependent apoptosis, and (b) suppression of prosurvival/antiapoptotic factors such as HER-1, HER-2, Akt, ERK, cellular FLICE-inhibitory protein (c-FLIP), and B-cell lymphoma 2 (Bcl-2), and survivin [13,18-24]. Additionally, α-TEA has been shown to stimulate antitumor immune responses [25].

Data presented here show that α-TEA circumvents TAMR in the presence of TAM via activation of endoplasmic reticulum stress-mediated DR5-dependent proapoptotic signaling and disruption of cholesterol-rich microdomains, leading to downregulation of prosurvival pathways.

## Materials and methods

### Chemicals

α-TEA (F.W. = 488.8) was prepared in our laboratory as previously described [16]. Tamoxifen was purchased from Calbiochem (La Jolla, CA). Filipin, methyl-β-cyclodextrin (MβCD) and cholesterol were purchased from Sigma (St. Louis, MO). Dialkylindocarbocyanine (DilC-16) was purchased from Molecular Probes (Eugene, OR).

### Cell culture and treatments

TAM-sensitive MCF-7/parental (MCF-7/TAMS) and acquired tamoxifen-resistant MCF-7 (MCF-7/TAMR) cells were a gift from Dr. Linda A. deGraffenried (University of Texas at Austin). MCF-7/TAMS cells were cultured as previously described [23]. MCF-7/TAMR cells were grown in phenol-red-free improved modified Eagle's medium (IMEM) with 10% charcoal stripped (steroid-depleted) serum supplemented with TAM (10^-7 ^*M*). [Note: TAM is required to maintain the TAMR phenotype of MCF-7/TAMR cells in culture]. Three days before treatment, cells were grown in phenol-red-free IMEM with 10% charcoal-stripped serum supplemented with 17-β-estradiol (10^-9 ^*M*) for MCF-7/TAMS and TAM (10^-7 ^*M*) for MCF-7/TAMR. Clone 18 MCF-7 cells overexpressing HER-2 (MCF-7/HER-2) that exhibit a TAMR phenotype [8] and their vector control (MCF-7/Neo) cells (gifts from Dr. Mien-Chie Hung, MD Anderson, Houston, TX) were cultured under the same condition as MCF-7/TAMS. During treatments, serum was reduced to 2% without TAM. Cells were treated with various concentrations of TAM, α-TEA, or vehicle (VEH) control. Neither TAM nor α-TEA is water soluble, so they are solubilized in ethanol before addition to media and an ethanol control (called vehicle), consisting of the highest concentration of ethanol used for solubilization in a given experiment, was included as a control.

### Western blot analyses

Western blot analyses were conducted as described previously [22]. Antibodies to the following proteins were used: poly (ADP-ribose) polymerase (PARP), c-FLIP, CHOP, glucose-regulated protein (GRP-78), pERK, total ERK (tERK), and pJNK (Santa Cruz Biotechnology, Santa Cruz, CA), Bcl-2, caspase-8, caspase-9, DR5, phospho-HER-1 (pHER-1), total HER-1 (tHER-1), phospho-HER-2 (pHER-2), total HER-2 (tHER-2), pER-α (Ser-118), pER-α (Ser-167), total ER-α (tER-α), pAkt (Ser-473), total Akt (tAkt), and glyceraldehyde-3-phosphate dehydrogenase (GAPDH) (Cell Signaling Technology, Beverly, MA).

### RNA interference

A scrambled RNA duplex that does not target any known gene was used as the nonspecific negative control for RNA interference (referred to as control siRNA). Transfection of MCF-7/TAMR cells with siRNAs to DR5, CHOP, JNK, Akt-1, c-FLIP, or control (Ambion, Austin, TX) was performed as previously described [22].

### Quantification of apoptosis

Apoptosis was quantified with the Annexin V-FITC/PI assay (Invitrogen, Carlsbad, CA) by following manufacturer's instructions. Data were analyzed by using CellQuest software (BD Biosciences, San Jose, CA).

### Staining with fluorescent-labeled DilC-16 and Filipin

Fluorescein-labeled lipid analogue DilC-16, a lipid microdomain marker [26], and fluorescein-labeled Filipin, a cholesterol marker [27], were used to determine the presence of cholesterol-enriched lipid microdomains. For DilC-16 staining, cells were trypsinized and washed with phosphate-buffered saline (PBS) and stained with DilC-16 (5 μ*M*) for 15 minutes at room temperature. For filipin staining, cells were trypsinized, washed with PBS, fixed with 3% paraformaldehyde for 30 minutes at room temperature, rinsed with PBS, and incubated with 1.5 mg/ml glycine in PBS to quench the paraformaldehyde. Cells were then stained with fluorescein-labeled filipin (50 μg/ml) for 1 hour at room temperature and washed with PBS. Cells were viewed by using a fluorescence microscope with TRITC filter setting for DiLC-16 staining and UV filter setting for filipin staining, respectively.

### Statistical analysis

One-way analysis of variance (ANOVA) followed by Tukey test was used for comparison of more than two treatments to determine statistical differences. Differences were considered statistically significant at *P *< 0.05.

## Results

TAMR cells in comparison with TAMS cells constitutively express higher levels of prosurvival mediators and cholesterol-enriched lipid microdomains

**Figure 1:**
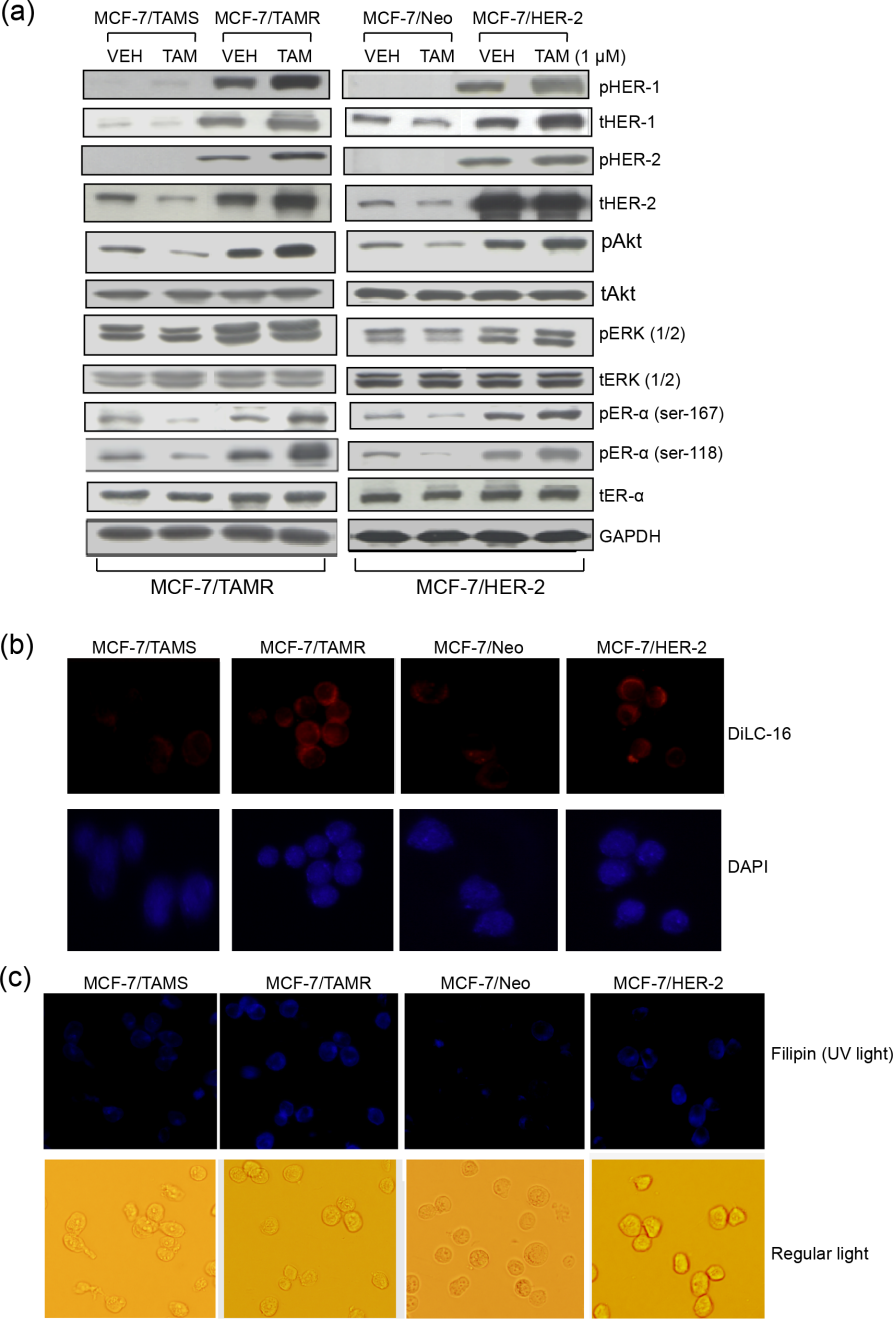
**TAMR cells in comparison to TAMS cells constitutively express higher levels of prosurvival mediators and cholesterol-rich lipid microdomains**. **(a) **TAMR cell lines MCF-7/TAMR and MCF-7/HER-2, as well as TAMS parental MCF-7/TAMS and MCF-7/Neo cells, were treated with TAM at 1 μ*M *or VEH (ethanol) in steroid-depleted media containing 17β-estradiol (10^-9 ^*M*) for 2 days. Molecular profile of prosurvival mediators was determined with Western blot analyses. (pHER-1/tHER-1 and pHER-2/tHER-2 were not detected in the MCF-7/TAMS and MCF-7/Neo cells). **(b) **The expression of lipid microdomains was determined by staining the cells with fluorescein-tagged DiLC16 lipid-raft marker and viewed with a fluorescence microscope. **(c) **The expression of cholesterol-rich lipid microdomains was determined by staining cells with fluorescein-labeled filipin, a cholesterol marker, and viewed by using a fluorescence microscope. **(a-c) **Data are representative of a minimum of three independent experiments. DiIC-16, dialkylindocarbocyanine; HER, human epidermal growth factor; MCF-7/HER-2, Clone 18 MCF-7 cells overexpressing HER-2; MCF-7/TAMR, acquired tamoxifen-resistant MCF-7; MCF-7/TAMS, TAM-sensitive MCF-7/parental; pHER-1, phosphorylated-HER-1; pHER-2, phosphorylated-HER-2; TAM, tamoxifen; TAMR, tamoxifen resistant; TAMS, tamoxifen sensitive; VEH, vehicle control.

Total and phosphorylated protein profiles of prosurvival mediators in both *de novo *and acquired TAMR cell lines (MCF-7/HER-2 and MCF-7/TAMR, respectively), cultured with and without TAM, in comparison with their parental TAMS cells, were determined by Western blot analyses. Growth-factor receptors HER-1 and HER-2 (phosphorylated form as well as total protein) and their downstream mediators pAkt and pERK1/2, as well as pER-α (Ser-167 and Ser-118) are expressed at markedly higher levels in TAMR cells in comparison with their parental TAMS cells (Figure 1a). pHER-1 and pHER-2 were below levels of detection in the TAMS cell lines either with or without TAM treatment (Figure 1a). As expected, TAM treatment reduced levels of activated Akt (pAkt) and activated ER-α (pER-α Ser-167 and Ser-118) in both TAMS cell lines, but had the opposite effect on the TAMR cells, measurably enhancing activated pAkt and pER-α (Ser-167 and Ser-118) in TAMR cells (Figure 1a). These data document that prosurvival signaling is constitutively highly expressed in TAMR cells in comparison with TAMS cells, and that TAM treatment differentially affects prosurvival signaling between TAMS and TAMR cells; TAM downregulates prosurvival mediators in TAMS cells and increases them in TAMR cells. Furthermore, both TAMR cell lines express higher levels of the fluorescent lipid analogue DilC-16, a marker of lipid microdomains, and fluorescein-labeled filipin, a cholesterol marker, as viewed by using a fluorescence microscope in comparison with TAMS cells (Figure 1b and 1c), suggesting that TAMR cells constitutively express higher levels of cholesterol-enriched lipid rafts that are supportive of prosurvival signaling.

### MβCD plus TAM treatment circumvents TAMR via induction of apoptosis and suppression of proliferation/survival signaling

**Figure 2:**
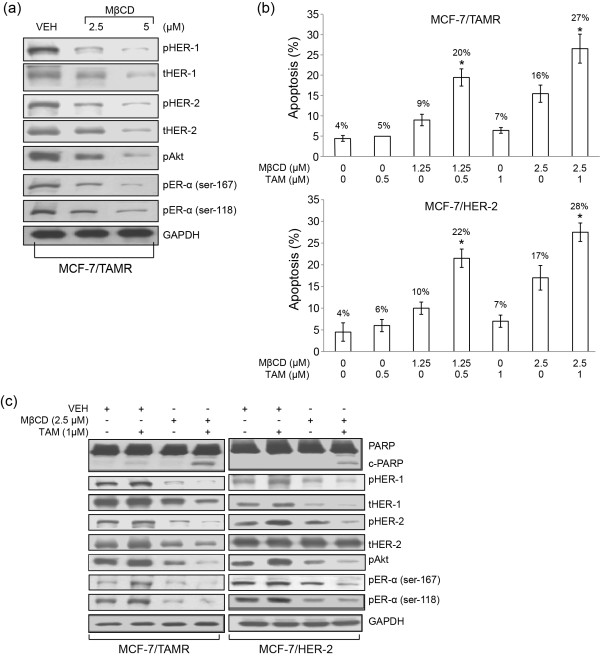
**Methyl-β-cyclodextrin (MβCD), a known cholesterol-rich membrane domain disruptor, reduces levels of prosurvival mediators and, in combination with TAM, induces apoptosis and reduces levels of prosurvival mediators**. **(a) **MCF-7/TAMR cells were treated with MβCD at 2.5 and 5.0 μ*M *for 1 day. Protein levels of prosurvival signaling mediators were determined with Western blot analyses. **(b) **MCF-7/TAMR and MCF-7/HER-2 cells were treated with TAM at 0.5 or 1 μ*M *with and without MβCD at 1.25 or 2.5 μ*M *for 1 day. Apoptosis was determined with Annexin V/PI. **(c) **Western blot analyses were performed to determine the prosurvival signaling mediators in both TAMR cell lines treated separately and in combination with 1 μ*M *TAM and 2.5 μ*M *MβCD for 1 day. Data in **(a) **and **(c) **are representative of two individual experiments. Data in **(b) **are depicted as mean ± SD of three individual experiments. *Significantly different in comparison with TAM or MβCD treatment alone; *P *< 0.05. TAM, tamoxifen; TAMR, tamoxifen resistant; MCF-7/HER-2, clone 18 MCF-7 cells overexpressing HER-2; MCF-7/TAMR, acquired tamoxifen-resistant MCF-7.

To determine whether cholesterol-rich lipid microdomains play a critical role in elevated expression of prosurvival mediators in TAMR cells, TAMR cells were cultured with the cholesterol-depleting agent MβCD followed by analyses of proliferation/survival mediators. MβCD at 2.5 and 5 μ*M *suppressed levels of total and pHER-1 and pHER-2, and decreased levels of pAkt and pER-α (Ser-118 and Ser-167) in MCF-7/TAMR cells (Figure 2a). MβCD at 1.25 or 2.5 μ*M *in the presence of 0.5 or 1 μ*M *TAM acted cooperatively to induce apoptosis significantly in both MCF-7/TAMR and MCF-7/HER-2 cells in comparison with single treatments, as determined with Annexin V-FITC/PI analyses (Figure 2b) and cleavage of PARP, an indicator of apoptosis (Figure 2c). Furthermore, although 1 μ*M *TAM treatment produced a trend toward increased levels of proliferation/survival mediators, MβCD alone produced a trend of decreased expression of proliferation/survival mediators, and the combination of MβCD + TAM acted cooperatively to produce the most marked reduction in proliferation/survival mediators (Figure 2c), indicating that MβCD restores TAM sensitivity. Taken together, these data demonstrate that MβCD disruption of cholesterol-rich lipid microdomains circumvents TAMR when combined with TAM via suppression of prosurvival signaling and induction of apoptosis, providing additional support that cholesterol-enriched lipid microdomains participate in TAM resistance via enhancing proliferation/prosurvival signaling in TAMR cells.

### α-TEA cooperates with TAM to induce apoptosis in TAMR cell lines

**Figure 3:**
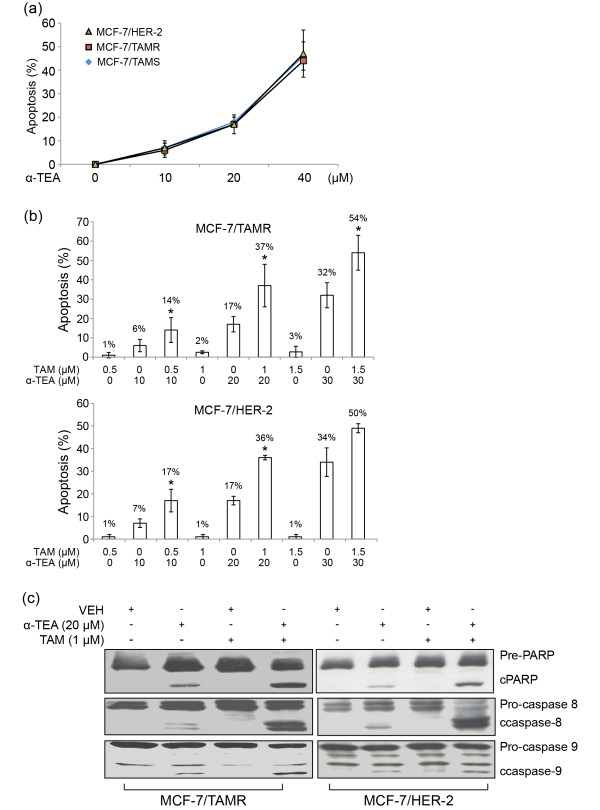
**α-TEA alone and in combination with TAM induces apoptosis in TAMR cell lines.(a) **TAM-sensitive MCF-7/parental cells and TAMR MCF-7/TAMR and MCF-7/HER-2 cells were treated with 10, 20, and 40 μ*M *α-TEA for 1 day. Apoptosis was determined with Annexin V/PI. **(b) **MCF-7/TAMR and MCF-7/HER-2 cells were treated with 10 or 20 or 30 μ*M *α-TEA, 0.5 or 1 or 1.5 μ*M *TAM, and in combination for 1 day. Apoptosis was determined with annexin V/PI/FACS assay. **(c) **MCF-7/TAMR and MCF-7/HER-2 cells were treated separately and in combination with 20 μ*M *α-TEA and 1 μ*M *TAM for 1 day. Western blot analyses were performed to determine cleaved (c-) forms of caspases-8, 9, and PARP. Data in **(a) **and **(b) **are depicted as mean ± SD of three individual experiments. *Significantly different in comparison with TAM and α-TEA alone; *P *< 0.05. Data in **(c) **are representative of three individual experiments. α-TEA, RRR-α-tocopherol ether-linked acetic acid analogue; HER-2, epidermal growth-factor receptor-2 ErbB-2; TAM, tamoxifen; TAMR, tamoxifen resistant; MCF-7/HER-2, clone 18 MCF-7 cells overexpressing HER-2; MCF-7/TAMR, acquired tamoxifen-resistant MCF-7.

α-TEA induces apoptosis in a dose-dependent manner in both TAMR and TAMS cells (Figure 3a). α-TEA treatment of MCF-7/TAMR and MCF-7/HER-2 at 10, 20, and 40 μ*M *significantly induced apoptosis in comparison with VEH control. As expected, TAM induced apoptosis only in TAM-sensitive MCF-7/parental cells, and not in either of the TAM-resistant cell lines (neither TAMR MCF-7/TAMR nor MCF-7/HER-2 cells (data not shown). To determine whether TAM can act cooperatively with α-TEA to trigger TAM-resistant cells to undergo apoptosis, we examined the combination effect of three nonapoptotic doses of tamoxifen (0.5, 1, and 1.5 μ*M*) with three sub-half-maximal effective concentration (EC_50_) apoptotic doses of α-TEA (10, 20, and 30 μ*M*) on induction of apoptosis. The combination of α-TEA at 10, 20, and 30 μ*M *plus TAM at 0.5, 1, and 1.5 μ*M*, respectively, significantly increased the levels of apoptosis (Figure 3b) and cleaved PARP (c-PARP) (Figure 3c) in MCF-7/TAMR and MCF-7/HER-2 cells in comparison with VEH control and single treatments.

We determined the proapoptotic effect obtained with TAM and α-TEA combination by using the CalcuSyn (Biosoft, Manchester, UK) software package, which is designed to calculate combination indexes (CIs) by using the Chou-Talalay method for drug-combination efficacy based on the median-effects equation [28]. CI values of 0.59 ± 0.00 and 0.81 ± 0.13 indicated synergistic actions of α-TEA + TAM (20:1 ratio) on induction of apoptosis in MCF-7/TAMR and MCF-7/HER-2 cell lines, respectively (Table 1). The cooperative proapoptotic actions of the combination of α-TEA plus TAM were further confirmed by measurement of increased levels of cleaved caspases-8 and -9 (c-caspase-8 and -9) (Figure 3c), suggesting that the combination of α-TEA + TAM induces caspase-8 and -9 mediated apoptosis in both TAMR cell lines.

**Table 1:** Combination index of apoptosisa

	α-TEA:TAM	CI^c^				
	**Ratio^b^**	**ED_50_**	**ED_75_**	**ED_90_**	**Mean + SD^d^**	
MCF-7/TAMR	20:1	0.59	0.59	0.59	0.59 ± 0.00	Synergism^a^
MCF-7/HER-2	20:1	0.68	0.80	0.94	0.81 ± 0.13	Synergism^a^

^a^MCF-7/TAMR and MCF-7/HER-2 human breast cancer cells were treated with different concentrations of α-TEA and TAM alone and in combinations for 24 hours. Apoptosis was determined as described in Material and Methods. ^b^The ratio for concentrations used in combination treatments were determined from data in Figure 3b and c. ^c^For each combination treatment, a combination index (CI) was calculated by using methods as described previously and by using the commercially available software (CalcuSyn; Biosoft, Manchester, United Kingdom). ^d^The mean ± standard deviation (SD) is calculated from the CI values of effective dosages (EDs) that produced 50%, 75%, and 90% apoptosis. ^e^CI values <1.0 indicate synergism; CI values of 1.0 indicate additive effect; and CI values >1.0 indicate antagonism. α-TEA, RRR-α-tocopherol ether-linked acetic acid analogue; MCF-7/HER-2, clone 18 MCF-7 cells overexpressing HER-2; MCF-7/TAMR, acquired tamoxifen-resistant MCF-7; mER, membrane estrogen receptor; TAM, tamoxifen.

### Combination of α-TEA + TAM acted cooperatively to induce endoplasmic reticulum stress-mediated apoptosis

**Figure 4:**
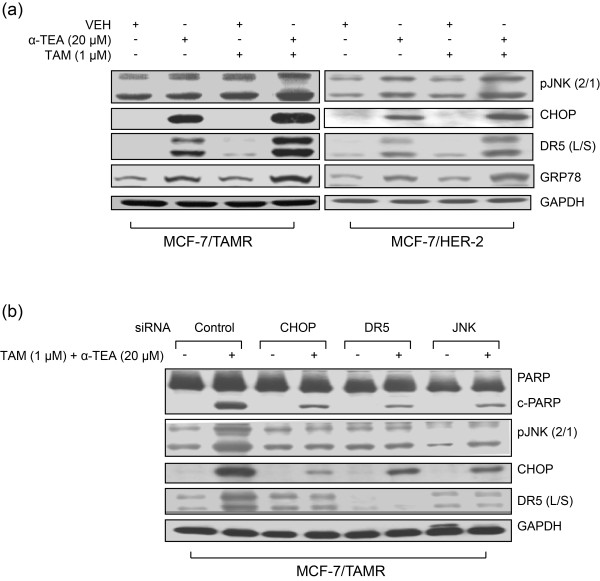
**α-TEA alone and in combination with TAM induces biomarkers of endoplasmic reticulum stress, and siRNA knockdowns show necessary roles for CHOP, DR5, and JNK**. **(a) **Western immunoblot analyses, using aliquots of cell lysates from cells treated with 20 μ*M *α-TEA and 1 μ*M *TAM in Figure 3b, were performed to assess pJNK2/1, CHOP, and DR5 (L/S) protein levels, as well as endoplasmic reticulum stress marker GRP78 protein expression with GAPDH as loading control. **(b) **MCF-7/TAMR cells transfected with siRNAs to CHOP, DR5, and JNK as well as control siRNA (labeled Control) were treated with TAM (1 μ*M*) + α-TEA (20 μM) for 1 day. Western immunoblot analyses were performed to determine degree of apoptosis, as measured by cleaved PARP (cPARP), pJNK2/1, CHOP, and DR5 (L/S) protein levels, with GAPDH serving as the loading control. Data from **(a) **and **(b) **are representative of three individual experiments. CHOP, Ccaat-enhancer-binding protein (C/EBP) homologous protein; DR5 (L/S), death receptor 5 long/short; GAPDH, glyceraldehyde-3-phosphate dehydrogenase; GRP78, glucose-regulated protein-78; JNK, c-Jun N-terminal kinase; MCF-7/TAMR, acquired tamoxifen-resistant MCF-7; PARP, poly (ADP-ribose) polymerase; siRNA, small interfering RNA; TAM, tamoxifen; TAMR, tamoxifen resistant; α-TEA, RRR-α-tocopherol ether-linked acetic acid analogue.

Because α-TEA has been shown to induce endoplasmic reticulum stress [18], we wanted to explore the possibility that α-TEA + TAM were inducing endoplasmic reticulum stress-mediated apoptosis. Combination of 20 μ*M *α-TEA + 1 μ*M *TAM induced increased levels of endoplasmic reticulum stress-associated proapoptotic factors, pJNK (2/1), CHOP, and DR5 long and short (L/S) and endoplasmic reticulum stress marker GRP78 in both TAMR cell lines (Figure 4a). siRNAs to CHOP, DR5, and JNK blocked the ability of the combination treatment to induce apoptosis, as determined by the absence of cleaved PARP and blockage of combination treatment-induced increases in pJNK (2/1), CHOP, and DR5 (L/S) in the MCF-7/TAMR cell line (Figure 4b), indicating that the combination of α-TEA + TAM enhances α-TEA-induced endoplasmic reticulum stress-mediated apoptosis, which involves JNK/CHOP/DR5.

### Combination of α-TEA + TAM circumvents TAMR via cooperatively suppressing prosurvival/antiapoptotic factors

**Figure 5:**
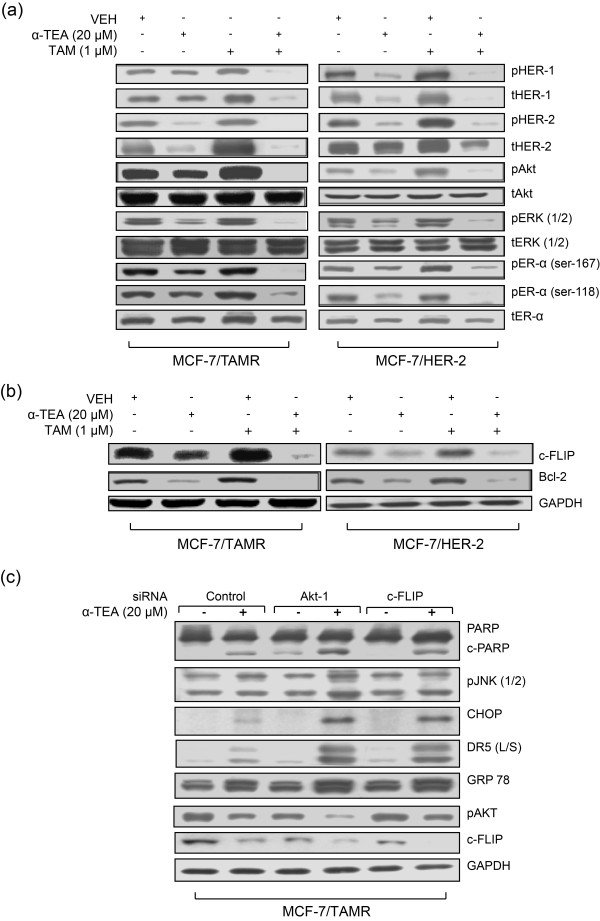
**Combination of α-TEA + TAM acts cooperatively to reduce prosurvival signaling in TAMR cells**. **(a, b) **Western immunoblot analyses using aliquots of cell lysates from treated cells in Figure 3b were performed to assess prosurvival signaling mediators **(a) **and to assess antiapoptotic factors c-FLIP and Bcl-2 protein expression **(b). (c) **MCF-7/TAMR cells transfected with siRNAs to Akt-1 or c-FLIP, as well as control siRNA (labeled Control), were treated with α-TEA (20 μ*M*) for 1 day. Western immunoblot analyses were performed to determine PARP, pJNK2/1, CHOP, DR5 (L/S), GRP 78, pAKT, and c-FLIP protein levels, with GAPDH serving as loading control. **(a-c) **Data are representative of three individual experiments. α-TEA, RRR-α-tocopherol ether-linked acetic acid analogue; Bcl-2, B-cell lymphoma 2; c-FLIP, cellular FLICE-inhibitory protein; CHOP, Ccaat-enhancer-binding protein (C/EBP) homologous protein; DR5 (L/S), death receptor 5 long/short; GAPDH, glyceraldehyde-3-phosphate dehydrogenase; GRP78, glucose-regulated protein-78; MCF-7/TAMR, acquired tamoxifen-resistant MCF-7; pJNK, phosphorylated-c-Jun N-terminal kinase; siRNA, small interfering RNA; TAM, tamoxifen; TAMR, tamoxifen resistant.

As shown in Figure 1a, TAM induces the expression of prosurvival mediators HER-1 and HER-2 (total and phosphorylated forms), as well as pAkt, pERK2/1, and pER-α (Ser 167 and 118) in TAMR cells. Importantly, α-TEA alone and, even more markedly, when in combination with TAM, reduced the expression of these proliferation/survival mediators (Figure 5a), indicating that α-TEA restores TAM sensitivity in TAMR cells by downregulating survival factors. Moreover, TAM alone induced increased levels of antiapoptotic factors c-FLIP and Bcl-2 (Figure 5b), suggesting that these antiapoptotic factors may be mediated by prosurvival signaling. Again, the combination of α-TEA + TAM acted cooperatively to suppress these antiapoptotic factors (Figure 5b).

In an effort to understand how TAM cooperates with α-TEA to induce endoplasmic reticulum stress and endoplasmic reticulum stress-mediated JNK/CHOP/DR5, we knocked down Akt-1 and c-FLIP by using siRNA to examine the impact on α-TEA-mediated upregulation of JNK1/2, CHOP, DR5, and GRP-78 protein levels. siRNAs to Akt-1 and c-FLIP enhanced α-TEA-induced apoptosis, as detected by PARP cleavage (Figure 5c), as well as enhanced the α-TEA ability to increase protein levels of JNK1/2, CHOP, DR5(L/S), and GRP-78 (Figure 5c). siRNA to Akt-1 reduced pAkt levels and reduced c-FLIP expression, and the combination of α-TEA + siRNA to Akt-1 acted cooperatively to suppress pAkt further and to reduce c-FLIP expression (Figure 5c). siRNA to c-FLIP reduced c-FLIP protein levels, but not pAkt, and acted cooperatively with α-TEA to reduce further the c-FLIP expression as well as to reduce pAkt levels (Figure 5c). These data suggest that c-FLIP is regulated, at least in part, by Akt-1, and downregulation of Akt/c-FLIP contributes to the α-TEA ability to upregulate pJNK, CHOP, DR5, and GRP78. Taken together, data presented in Figure 5 demonstrate that the combination of α-TEA + TAM acts cooperatively to suppress markedly both prosurvival and antiapoptotic signaling mediators.

### Reductions in cholesterol-rich lipid-raft domains are involved in α-TEA + TAM circumvention of TAMR

**Figure 6:**
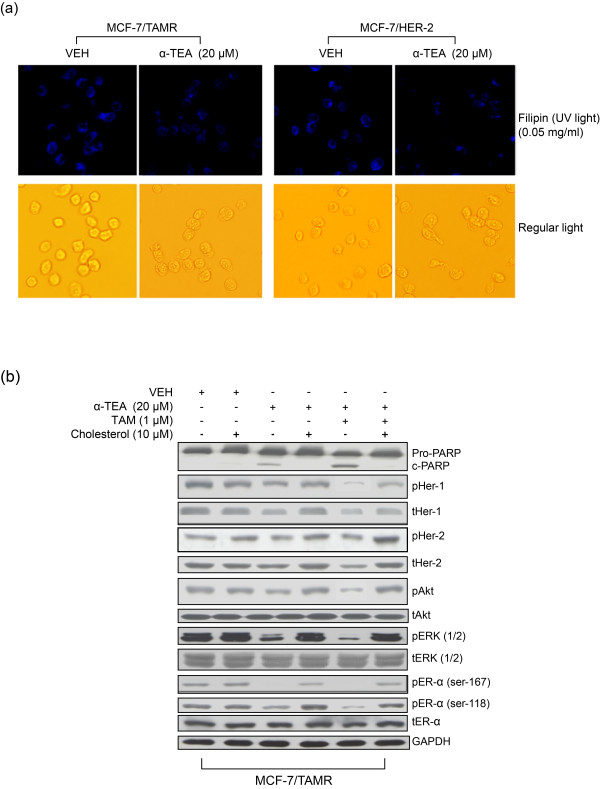
**Cholesterol-rich lipid microdomains are involved in α-TEA + TAM circumvention of TAMR**. **(a) **MCF-7/TAMR and MCF-7/HER-2 cells were treated with α-TEA at 20 μ*M *for 15 hours. Identification of cholesterol-rich microdomains was determined by staining the cells with fluorescein-tagged cholesterol marker Filipin and images viewed by using a fluorescence microscope. **(b) **MCF-7/TAMR cells were pretreated with 10 μ*M *exogenous cholesterol for 2 hours, followed by treatment with 20 μ*M *α-TEA or 20 μ*M *α-TEA plus 1 μ*M *TAM for l day. Western blot analyses were performed to determine treatment effects on prosurvival signaling mediators and PARP cleavage. **(a, b) **Data are representative of three independent experiments. α-TEA, RRR-α-tocopherol ether-linked acetic acid analogue; MCF-7/HER-2, clone 18 MCF-7 cells overexpressing HER-2; MCF-7/TAMR, acquired tamoxifen-resistant MCF-7; PARP, poly (ADP-ribose) polymerase; TAM, tamoxifen; TAMR, tamoxifen resistant.

Cholesterol-rich lipid microdomains support cell proliferation and cell survival. As detected by staining cells with the cholesterol marker filipin, treatment with α-TEA in comparison with VEH control produced reductions in cholesterol-rich microdomains (Figure 6a). Pretreatment of MCF-7/TAMR cells with 10 μ*M *exogenous cholesterol, an established method for enhancing cholesterol-rich microdomains [29] for 2 hours blocked the ability of both α-TEA alone and the combination of α-TEA + TAM to induce apoptosis, as detected by PARP cleavage (Figure 6b); as well as to decrease protein levels of prosurvival signaling mediators (Figure 6b). These data suggest that cholesterol-rich lipid microdomains are important for α-TEA + TAM circumvention of TAMR.

## Discussion

Acquired and *de novo *tamoxifen resistance are major barriers for successful application of tamoxifen in the clinic. Data reported here document that TAMR cells constitutively express highly elevated growth-factor signaling mediators that can be depleted by reducing cholesterol-rich microdomains and that α-TEA, a small bioactive lipid, in combination with TAM, restores TAM sensitivity to TAMR cells via suppression of TAMR proliferation/survival mediators and induction of cell death by apoptosis. Novel findings from these studies are as follows: (a) TAMR cells express higher levels of cholesterol-rich lipid microdomains than do TAMS cells; (b) disrupting cholesterol-rich lipid microdomains with the cholesterol-depleting agent MβCD suppressed TAMR prosurvival signaling and induced apoptosis when combined with TAM; (c) treating TAMR cells with the unique anticancer agent α-TEA alone reduced cholesterol-rich lipid microdomains, reduced levels of constitutively expressed pro-proliferation/prosurvival signaling mediators, and led to apoptosis via endoplasmic reticulum stress-mediated JNK/CHOP/DR5 signaling; (d) the combination of α-TEA + TAM had the best impact on circumventing TAMR via decreased expression of prosurvival/antiapoptotic mediators and induction of endoplasmic reticulum stress-mediated JNK/CHOP/DR5 proapoptotic mediators; and (e) suppression of constitutively expressed pAkt or c-FLIP in cells by siRNA enhanced α-TEA-induced apoptosis; as well as endoplasmic reticulum stress-mediated JNK/CHOP/DR5 signaling, indicating an important role for crosstalk between prosurvival Akt/antiapoptotic c-FLIP and the pro-death endoplasmic reticulum stress pathway.

Taken together, our data for the first time demonstrate that reducing cholesterol-rich lipid microdomains is a promising strategy for circumventing TAMR in human breast cancer cells and that the combination of α-TEA + TAM is a potentially beneficial regimen for preventing and circumventing TAMR in human breast cancer.

It is well established that TAMR is caused by overexpression of receptor tyrosine kinases (RTKs) proteins such as HER-1 and HER-2, which enhance RTK crosstalk with membrane-associated estrogen receptor, resulting in ER-independent and -dependent cell proliferation and survival via their downstream mediators Akt and ERK. Akt and ERK regulate multiple prosurvival factors; as well as activate nuclear ER-α via phosphorylation of ER-α at Ser-118 by ERK and Ser-167 by Akt [7,30]. Furthermore, it is well established that TAM acts as an agonist in TAMR cells stimulating prosurvival signaling [7,30]. Thus, the combination of constitutively highly expressed prosurvival mediators and TAM, acting as an agonist rather than antagonist, are key molecular features of both *de novo *and acquired ER+ TAMR cells. Targeting various components in this highly amplified prosurvival signaling context, such as HER-1, HER-2, Akt, or mTOR, by using chemical inhibitors or neutralizing antibodies has been reported to circumvent TAMR [31-34]. However, targeting single components, such as HER-1 or HER-2, eventually causes resistance [35,36]. Therefore, alternative approaches are required to circumvent TAMR.

Cholesterol-enriched lipid microdomains have been shown to provide platforms for crosstalk among growth factors, their receptors, and downstream mediators, leading to activation of prosurvival signaling [10,11,37]. It has also been reported that cholesterol-enriched lipid microdomains participate in crosstalk between mER and growth-factor receptors [38]. Furthermore, cholesterol-rich membrane rafts have been hypothesized to provide privileged sites for nongenomic hormone signaling in prostate cancer cells, which could stimulate cell proliferation [39]. Therefore, disrupting cholesterol-rich lipid microdomains holds promise as a strategy for circumventing TAMR via downregulation of prosurvival signaling. However, little information exists on the role of cholesterol-rich lipid microdomains in TAMR. In this study, we examined whether alterations in the cholesterol content of lipid rafts in TAMR cell membranes affected cell-survival mediators. Here, for the first time, we report that TAMR cells expressed high levels of cholesterol-rich lipid microdomains, and that MβCD, a cholesterol-depleting agent that is used in research to disrupt lipid rafts, suppresses TAMR prosurvival signaling and circumvents TAMR when combined with TAM via restoration of TAM sensitivity and induction of apoptosis. These results implicate the necessity of cholesterol-enriched domains in survival of TAMR cells and suggest that agents that can disrupt cholesterol-enriched domains have potential as a promising strategy to circumvent TAMR when combined with TAM in ER+ breast cancer. Based on data presented here, α-TEA is one such agent.

α-TEA exerts its anticancer actions via activation of proapoptotic pathways and suppression of prosurvival pathways. However, molecular details of how α-TEA affects these prosurvival/antiapoptotic factors are not fully understood. Previously, we reported that α-TEA downregulates phosphatidylinositol-3-kinase (PI3K)/Akt/ERK pathways via JNK-mediated downregulation of insulin-receptor substrate (IRS-1) [20]. Data presented here suggest that disruption of cholesterol-rich lipid microdomains may be another mechanism of α-TEA action. The data to support this notion come from data presented here showing the following: (a) α-TEA disrupts cholesterol-rich lipid microdomains; (b) addition of exogenous cholesterol to enrich lipid microdomains further, blocks the ability of α-TEA to suppress prosurvival mediators; and (c) α-TEA acts in a cooperative manner with MβCD, a cholesterol disruptor, more markedly to suppress TAMR prosurvival signaling. How α-TEA disrupts cholesterol-rich lipid microdomains is not known. Because α-TEA has been reported to enhance ceramide accumulation in cellular membranes [40] and ceramide-enriched lipid microdomains have been reported to disrupt cholesterol lipid microdomains [41], one possibility is that α-TEA disrupts cholesterol-rich plasma membrane domains by increasing ceramide-rich lipid microdomains.

Mechanistically, both MβCD and α-TEA cooperated with TAM to suppress TAMR prosurvival signaling, leading to TAM-induced apoptosis. Both agents reduced cholesterol-rich lipid microdomains, which was demonstrated to be critical to both MβCD and α-TEA circumvention of TAMR. However, although both MβCD and α-TEA cooperated with TAM to induce apoptosis in TAMR cells the mechanisms are not the same. α-TEA + TAM induces endoplasmic reticulum stress-mediated JNK/CHOP/DR5 proapoptotic events, whereas MβCD + TAM did not induce endoplasmic reticulum stress (data not shown). In summary, α-TEA + TAM induces apoptosis not only via suppression of prosurvival pathways, but also via activation of endoplasmic reticulum stress-mediated pro-apoptotic events, demonstrating that the α-TEA + TAM combination is a unique regimen for circumvention of TAMR.

How TAM cooperatively acts with α-TEA to induce endoplasmic reticulum stress-mediated JNK/CHOP/DR5 is not entirely clear. One possibility is that crosstalk occurs between α-TEA downregulation of prosurvival signaling and induction of endoplasmic reticulum stress. Published data show that downregulation of c-FLIP can enhance the α-TEA-induced endoplasmic reticulum stress proapoptotic pathway via activation of caspase-8, because caspase-8 is involved in α-TEA-induced endoplasmic reticulum stress [18]. Data presented here show that siRNA to Akt-1 blocked c-FLIP protein expression, suggesting that Akt is an upstream mediator of c-FLIP. Furthermore, siRNA inhibition of either Akt-1 or c-FLIP enhanced α-TEA-induced endoplasmic reticulum stress and endoplasmic reticulum stress-mediated upregulation of JNK/CHOP/DR5, indicating that suppression of prosurvival mediators by TAM + α-TEA may enhance α-TEA-induced endoplasmic reticulum stress-mediated JNK/CHOP/DR5, at least in part, via downregulation of activated Akt, which subsequently downregulates c-FLIP.

**Figure 7:**
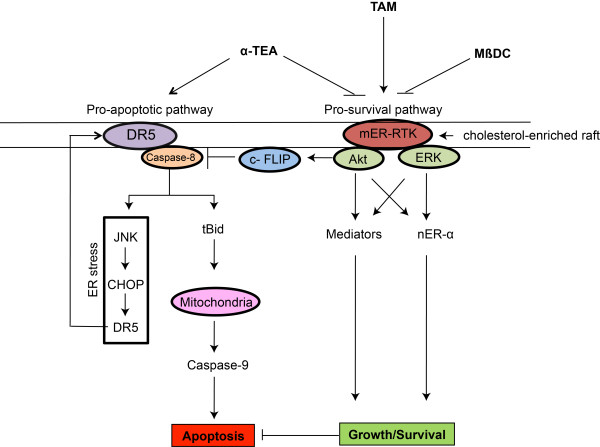
**Proposed signaling pathways modulated by α-TEA and TAM in TAMR cells**. Based on published data and data presented here, a schematic diagram of the actions of α-TEA, MβCD, and TAM on proapoptotic and prosurvival signaling mediators in TAMR cells is depicted. **(a) **Proapoptotic pathway affected by α-TEA and α-TEA + TAM: α-TEA triggers DR5 extrinsic death receptor-mediated caspase-8/tBid/Bax/mitochondria/caspase-9-dependent apoptosis and caspase-8-mediated endoplasmic reticulum stress-dependent upregulation of JNK/CHOP/DR5 positive-feedback loop. Combination of α-TEA + TAM enhances these apoptotic pathways via suppression of Akt-mediated c-FLIP. **(b) **Prosurvival pathways affected by α-TEA, MβCD, and TAM. Both α-TEA and MβCD disrupt cholesterol-rich lipid microdomains, leading to suppression of crosstalk between mER-α and RTKs, reduction of total and phosphoprotein levels of RTKs (HER-1 and HER-2), decreased levels of pAkt and pERK1/2, as well as decreased levels of estrogen receptor-α activity via downregulation of nuclear estrogen receptor-α (nER-α) phosphorylation mediated by Akt and ERK (Ser-118 and Ser-167). Both Akt and ERK mediated downstream prosurvival/antiapoptotic mediators, and nER-α promoted proliferation/survival and inhibited apoptosis. In contrast, TAM stimulates survival signaling in TAMR cells. **(c) **Prosurvival pathways affected by combination of α-TEA or MβCD + TAM: α-TEA or MβCD + TAM cooperatively act to suppress prosurvival signaling, indicating that either α-TEA or MβCD restores TAM sensitivity by converting the TAM prosurvival (agonistic) actions to antisurvival (antagonistic) actions. c-FLIP, cellular FLICE-inhibitory protein; CHOP, Ccaat-enhancer-binding protein (C/EBP) homologous protein; DR5 (L/S), death receptor 5 long/short; HER-1, epidermal growth factor receptor ErbB-1; HER-2, epidermal growth factor receptor-2 ErbB-2; JNK, c-Jun N-terminal kinase; MCF-7/HER-2, clone 18 MCF-7 cells overexpressing HER-2; MCF-7/TAMR, acquired tamoxifen-resistant MCF-7; mER, membrane estrogen receptor; MβCD, methyl-b-cyclodextrin; nER, nuclear estrogen receptor; pAkt, phosphorylated-Akt; pERK1/2, phosphorylated-extracellular signal-regulated kinases 1 and 2; RTKs, receptor tyrosine kinases; TAM, tamoxifen; TAMR, tamoxifen resistant; tBid, truncated Bid; α-TEA, RRR-α-tocopherol ether-linked acetic acid analogue.

Based on published data and data present here, a schematic diagram of the known actions of α-TEA, MβCD, and TAM on proapoptotic and prosurvival signaling in TAMR cells is depicted in Figure 7.

## Conclusions

In summary, α-TEA functions as a disruptor of cholesterol-rich lipid microdomains and an endoplasmic reticulum stress inducer in circumvention of TAMR. Although α-TEA can effectively induce TAMR cells to undergo apoptosis as a single agent, it acts cooperatively with TAM at lower dosages to activate endoplasmic reticulum stress-mediated proapoptotic events and suppresses the highly amplified prosurvival signaling inherent in TAMR cells. As a potent anticancer agent, α-TEA possesses several compelling features: (a) low toxicity to normal cells and tissues [12]; (b) dual anticancer actions (that is, suppresses prosurvival mediators and activates proapoptotic mediators); and (c) is effective against a wide range of cancer cell types with disparate molecular signatures [42]. Unlike single agents that target HER-1, HER-2, Akt, or mTOR, α-TEA can inhibit multiple prosurvival mediators via disruption of cholesterol-rich lipid microdomains and induce apoptosis. These unique features of α-TEA suggest great potential for use as a stand-alone adjuvant therapy or in combination with the adjuvant TAM for prevention and circumvention of TAMR in ER+ human breast cancers. Additionally, these data provide new knowledge about mechanisms of endocrine therapy resistance that may be useful in designing other agents for circumvention of TAMR.

## Abbreviations

α-TEA: RRR-α-tocopherol ether-linked acetic acid analogue; Bcl-2: B-cell lymphoma 2; c-FLIP: cellular FLICE-inhibitory protein; CHOP: Ccaat-enhancer-binding protein (C/EBP) homologous protein; CI: combination index; c-PARP: cleaved poly (ADP-ribose) polymerase; DAPI: 4'-6-diamidino-2-phenylindole; DiIC-16: dialkylindocarbocyanine; DR5: death receptor 5; DR5 (L/S): death receptor 5 long/short; EC_50_: half-maximal effective concentration; ED: effective dosages; ER: estrogen receptor; ER-α estrogen receptor-α ER+: estrogen receptor positive; FITC: fluorescein isothiocyanate; GAPDH: glyceraldehyde-3-phosphate dehydrogenase; GRP78: glucose-regulated protein-78; HER-1: epidermal growth factor receptor ErbB-1; HER-2: epidermal growth factor receptor-2 ErbB-2; IRS: insulin-receptor substrate; JNK: c-Jun N-terminal kinase; MβCD: methyl-β-cyclodextrin; MCF-7/HER-2: clone 18 MCF-7 cells overexpressing HER-2; MCF-7/TAMR: acquired tamoxifen-resistant MCF-7; MCF-7/TAMS: TAM-sensitive MCF-7/parental; mER: membrane estrogen receptor; mTOR: mammalian target of rapamycin; nER: nuclear estrogen receptor; pAkt: phosphorylated-Akt; PARP: poly(ADP-ribose) polymerase; PBS: phosphate-buffered saline; pERK1/2: phosphorylated-extracellular signal-regulated kinases 1 and 2; pER-α (Ser-167): phosphorylated form of estrogen receptor-α at serine-167; pER-α (Ser-118): phosphorylated form of estrogen receptor-α at serine-118; pHER-1: phosphorylated-HER-1; pHER-2: phosphorylated-HER-2; PI: propidium iodide; PI3K: phosphatidylinositol-3-kinase; pJNK: phosphorylated-c-Jun N-terminal kinase; pmTOR: phosphorylated mammalian target of rapamycin; RTKs: receptor tyrosine kinases; siRNA: small interfering RNA; TAM: tamoxifen; TAMR: tamoxifen resistant; TAMS: tamoxifen sensitive; tAkt: total Akt; tER-α total estrogen receptor-α tERK1/2: total extracellular signal-regulated kinases 1 and 2; tHER-1: total HER-1; tHER-2: total HER-2; tBid: truncated Bid; VEH: vehicle control.

## Competing interests

K Kline, BG Sanders, and W Yu are co-inventors on α-TEA patents, which are assigned to the Research Development Foundation (a nonprofit foundation that supports medical related research). A possibility exists that financial gain may be realized if α-TEA is successfully developed for clinical use. The authors declare that they have no competing interests.

## Authors' contributions

WY, BGS, and KK conceived and designed the study, analyzed the data, and drafted the manuscript. RT performed all the experiments, conceived and designed the study, analyzed the data, and drafted the manuscript. LAD provided original MCF-7/TAMR and TAMS cells. All authors read and approved the manuscript for publication.
